# Personalised computational cardiology: Patient-specific modelling in cardiac mechanics and biomaterial injection therapies for myocardial infarction

**DOI:** 10.1007/s10741-016-9528-9

**Published:** 2016-02-01

**Authors:** Kevin L. Sack, Neil H. Davies, Julius M. Guccione, Thomas Franz

**Affiliations:** 1 Division of Biomedical Engineering, Department of Human Biology, Faculty of Health Sciences, University of Cape Town, Private Bag X3, 7935 Observatory, South Africa; 2 Cardiovascular Research Unit, MRC IUCHRU, Chris Barnard Division of Cardiothoracic Surgery, University of Cape Town, Observatory, South Africa; 3 Department of Surgery, University of California at San Francisco, San Francisco, CA USA

**Keywords:** Cardiac disease, Finite-element method, Subject specific, Computational model, Ischaemic heart disease, Heart failure

## Abstract

Predictive computational modelling in biomedical research offers the potential to integrate diverse data, uncover biological mechanisms that are not easily accessible through experimental methods and expose gaps in knowledge requiring further research. Recent developments in computing and diagnostic technologies have initiated the advancement of computational models in terms of complexity and specificity. Consequently, computational modelling can increasingly be utilised as enabling and complementing modality in the clinic—with medical decisions and interventions being personalised. Myocardial infarction and heart failure are amongst the leading causes of death globally despite optimal modern treatment. The development of novel MI therapies is challenging and may be greatly facilitated through predictive modelling. Here, we review the advances in patient-specific modelling of cardiac mechanics, distinguishing specificity in cardiac geometry, myofibre architecture and mechanical tissue properties. Thereafter, the focus narrows to the mechanics of the infarcted heart and treatment of myocardial infarction with particular attention on intramyocardial biomaterial delivery.

## Introduction

Cardiovascular diseases are the single leading cause of death worldwide, accounting for 30 % of all human mortality [1]. Despite recent advances in pharmaceutical, surgical, device and tissue-engineered therapy strategies, cardiovascular diseases remain one of the most costly, common and deadly medical conditions. Since predicted mortality of cardiovascular diseases is projected to increase, it is expected to remain the leading cause of death globally [1, 2].

Computational models can provide a unique framework for assessing efficacy of therapy approaches with relatively low resources: Therapeutic parameters can be easily modified and assessed in multiple concurrent in silico experiments, and computational sensitivity studies are easily conducted to optimise treatment efficacy. Advancing research and technologies have sparked a great deal of interest in integrating FE models into the clinical environment. This is becoming more achievable each year, making it likely that computational models will serve as the first line of the screening for future therapies in the years to come [3].

**Fig. 1:**
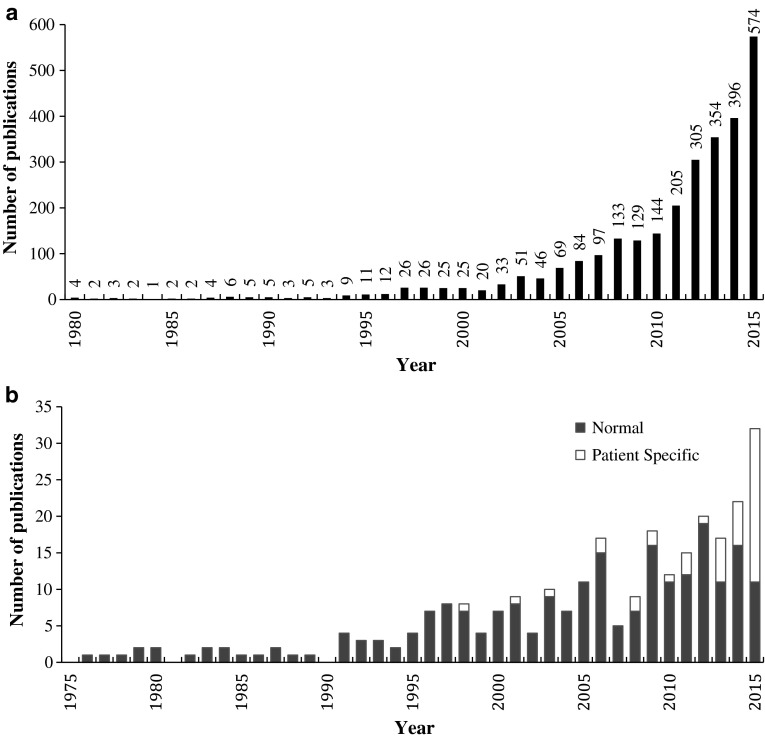
Number of yearly publications of peer-reviewed journal articles **a** with “patient-specific” or “subject-specific” contained in the title and **b** for finite-element-based studies focusing on cardiac ventricular mechanics. *Source*: Thomson Reuters ISI Web of Knowledge^®^ and PubMed^®^ databases, January 2016

Reliable computational models can also provide a richer source of information for clinical decision support and treatment planning. Patient- and subject-specific computational modelling has been increasing at an exponential rate (Fig. 1a), and sources of patient-specific genetic, anatomical and physiological information are already being incorporated in the clinical workflow [4–6].

This review aims at contextualising the advances and challenges of patient-specific computational modelling with particular focus on cardiac and infarct mechanics and the translation of therapeutic concepts, based on intramyocardial biomaterial injections, for the myocardial infarction (MI) and infarct-induced heart failure (HF). The developments in subject- and patient-specific modelling are detailed with focus on cardiac geometries, myofibre architecture and the constitutive properties of cardiac tissue. Thereafter, the focus narrows on computational modelling of infarct mechanics and therapies for MI, in particular intramyocardial biomaterial injection.

## Patient-specific modelling of cardiac mechanics

### Cardiac geometries

Anatomical simplification needs to balance model accuracy and computational demands. Until recently, this motivated the use of simplified left ventricular (LV) geometries introduced by various groups [7–9] as the primary computational tool for investigating cardiac mechanics. The use of patient-specific realistic geometries has, however, become prevalent in computational models (Fig. 1b). This shift towards realistic geometries is indicative of the goal to create more representative computational models for use in clinical decision support.

The first three-dimensional patient-specific computational geometry of a heart was introduced by Okajima et al. [10] to study electrical activation. It took over two decades for deformable computational finite-element (FE) approaches to incorporate realistic heart geometries [11]. Nielsen et al. [12] presented a realistic biventricular model that was novel for its accurate geometric description and definition of myofibre orientation. Stevens et al. [13] extended the model to account, in part, for the four valves. Since then, realistic geometrical models have become increasingly popular. The Living Heart Project recently developed the first full heart model that features a four-chamber human heart geometry with the four valves and the connecting large vessels [14].

To date, patient-specific cardiac geometries have been introduced as representative cases as proof of concept that a given computational approach can be applied in a patient-specific framework. Extending the concept to computational studies with a large number of patient-specific geometries could generate statistically meaningful results for a patient population. An alternative approach is to utilise a statistically averaged geometry that is representative of a patient population [15].

### Myofibre architecture

The myofibre orientation has a critical influence on cardiac mechanics and electrophysiology. However, the description of myofibre orientation is a highly intricate and sophisticated task, which has been the subject of substantial historical disagreement [16–18]. The intricacy stems from the complex multiscale branching and merging of cardiac myocytes at microscopic scale, creating anisotropy at tissue level that changes dramatically throughout the structure. An accurate numerical portrayal of the myofibre architecture needs to incorporate the one-dimensional directional tangent of the myofibre and the description of the fibre sheets [18–20] which influences both passive and active material behaviour.

In recent work, two methods are predominant in describing the myofibre orientation in patient-specific cardiac models: Rule-based reconstructions and fibre orientation derived from diffusion tensor magnetic resonance imaging (DTMRI). Rule-based approaches typically describe the fibre orientation analytically or through aggregated experimental data, whereby a dense fibre orientation field is constructed through interpolation functions [21, 22]. This has recently been cast in the form of a boundary value problem, whereby the fibre orientation is prescribed along the surfaces of the ventricular structure and solved for throughout the geometry [23]. Rule-based reconstructions can be advantageous in their application to highly irregular geometries and their efficient implementation. In DTMRI approaches, the myofibre orientation is calculated from the eigenvectors of diffusion tensors. Due to the challenges of in vivo cardiac DTMRI, this method is often limited to a single *post mortem* data set. In this case, the derived fibre orientations are projected (or mapped) onto other subject- or patient-specific geometries obtained from computed tomography (CT) or magnetic resonance imaging (MRI) [24, 25]. Toussaint et al. [26] recently captured in vivo patient-specific myofibre orientation data and integrated these data in other LV models of other patients. This approach involved diffeomorphic data transformations between a realistic geometry and the prolate spheroidal coordinate system.

Considering the sensitivity of FE predictions to variations in fibre orientation [27–29], it is preferable to incorporate patient-specific DTMRI data whenever possible. DTMRI is, however, still limited as the diffusion tensor characterises only the mean myofibre structure in a voxel volume. Improved accuracy may be achieved by increased spatial resolution of the DTMRI scan or by including a dispersion parameter accounting for the deviation of the fibre orientation within in a voxel. Whereas homogenous dispersion has been considered in modelling myocardial tissue [30], dispersion at voxel scale has not yet been considered. Toussaint et al. [26] in vivo DTMRI analysis is the most advanced approach at present. No computational models have investigated cardiac function using truly individual patient-specific fibre orientation.

### Constitutive properties

The first constitutive relationship for passive myocardial behaviour, in the form of an exponential strain energy formulation, has been credited to by Yuan-Cheng Fung [31–33]. The first invariant-based constitutive model was described by Humphrey and Yin [34], introducing an additional “fibre-specific” term to account for the material anisotropy, although limited to transverse isotropy [32]. Costa et al. [35] developed an extended orthotropic formulation featuring a fibre-specific coordinate system and principal material stiffness along the fibre, sheet and normal directions. With minor modifications, the formulations are commonly represented as strain energy density function [36, 37]:

1 $$\Psi = \frac{1}{2}\left( {{Ce}^{Q} - 1} \right) + A_{\text{incomp}} .$$

Here *Q* is a function of the material strains (usually Green–Lagrange), often given as

2 $$Q = b_{ff} E_{ff}^{2} + b_{ss} E_{ss}^{2} + b_{nn} E_{nn}^{2} + \frac{{b_{nf} }}{2}\left( {E_{nf}^{2} + E_{fn}^{2} } \right) + \frac{{b_{ns} }}{2}\left( {E_{ns}^{2} + E_{sn}^{2} } \right) + \frac{{b_{sf} }}{2}\left( {E_{sf}^{2} + E_{fs}^{2} } \right),$$

where *E*_*ij*_ are the components of the right Cauchy-Green deformation tensor in local fibre coordinates and *b*_*ij*_ are the corresponding material parameters. The strain energy function in the exponential form can also be constructed by considering the invariants *I*_*i*_ of the right Cauchy-Green strain tensor [38]:

3 $$\Psi = \frac{a}{2b}e^{{b\left( {I_{1} - 3} \right)}} + \mathop \sum \limits_{i = f,s} \frac{{a_{i} }}{{2b_{i} }}\left\{ {e^{{b_{i} \left( {I_{4i} - 1} \right)^{2} }} - 1} \right\} + \frac{{a_{fs} }}{{2b_{fs} }}\left\{ {e^{{b_{fs } I_{8fs}^{2} }} - 1} \right\} + A_{\text{incomp}} ,$$

The notation and material parameters detailed by Holzapfel and Ogden [38] have become the most widely used form of a passive material law for cardiac mechanics, often recalibrated with new material parameters [22, 39, 40]. The incompressibility of the material is handled through a penalty function *A*_incomp_, of which multiple variations exist, and which is often implemented through mixed formulation methods, splitting the deformation into isochoric and deviatoric components.

The incorporation of active tension into a mathematical description to capture the contractile behaviour of the heart is a significant task. The most common approach, introduced by Guccione et al. [41], relies on additive contribution of the active stress to the overall material stress, typically along the local fibre orientation. Active tension can be constructed using various physiologically meaningful parameters, which has been employed by many studies [14, 39, 42, 43]. Another emerging approach involves multiplicative decomposition of the tensor gradient of deformation [44–46], in a similar fashion to the theory of volumetric growth. Multiplicative decomposition is more mathematically robust, whereas the additive approach can capture physiological phenomena more meaningfully. The latter is due to a more flexible formulation that allows for parameter calibration on a tensor component level [44, 45]. Contractile material behaviour can be coupled within an electromechanical framework, whereby a more realistic excitation–contraction pattern is incorporated into the model. Over the last decade, this multi-physics coupling has been introduced with great success in various computational models [47–53].

It is impossible to meaningfully determine the three-dimensional patient-specific material properties from ventricular pressure–volume relationships alone [54]. To remedy this, the identification of suitable values for the constitutive parameters (sometimes called calibration) often utilises additional data. In vitro biaxial and shear stress–strain experiments [55, 56] on cardiac tissue have been used to calibrate numerous constitutive laws. The reliability of in vitro experiments may be questionable due to tissue damage or disruption in the process of extraction [57]. Inclusion of in vivo data in material calibration provides a more realistic mechanical environment for loading and deformation [58]. Obtaining these stress–strain data from magnetic resonance electrography (MRE) or tagged MRI provides additional advantages whereby patient-specific information can be captured into the constitutive law through optimisation approaches [24, 54, 59–61]. This approach has recently been extended to additionally estimate infarct material parameters [62, 63]. Considering the anatomical variability amongst patients, it becomes increasingly attractive to calibrate material laws for geometrically consistent in vivo data, i.e., local stress–strain data for the same geometry and fibre distribution—an inherently patient- or subject-specific process that can easily be adopted to handle cardiac pathologies, e.g., MI.

## Modelling of MI treatments

Noninvasive assessment of the heart after MI is essential for optimal treatment. Local wall stress, in particular, can be a key factor in assessing cardiac function and predict post-MI effects, yet wall stress cannot be measured systematically and quantitatively with clinical modalities [64]. Imaging techniques provide high accuracy information regarding the strain distribution in the heart, yet cannot provide local stress information. Laplace’s law, used to estimate cardiac wall stress, makes considerable assumptions with respect to the cardiac structure and provides considerably different results to anatomically accurate FE models [65].

FE models have been labelled as the most versatile approach for quantitatively predicting myocardial stress and strain distributions [66, 67]. The effects of MI on structure and function of the heart have received increasing attention for FE modelling. The understanding of the aforementioned is essential when developing a treatment to restore cardiac function and to attenuate adverse post-infarct remodelling. Simulating MI in silico allows the influence on heart to be directly quantified [46, 68–71], providing deeper insight into the underlying mechanisms involved. For example, by complementing a study on dosage efficacy of the ACE inhibitor ramipril, FE models showed that apical wall stress is an independent predictor of ventricular remodelling [72].

**Fig. 2:**
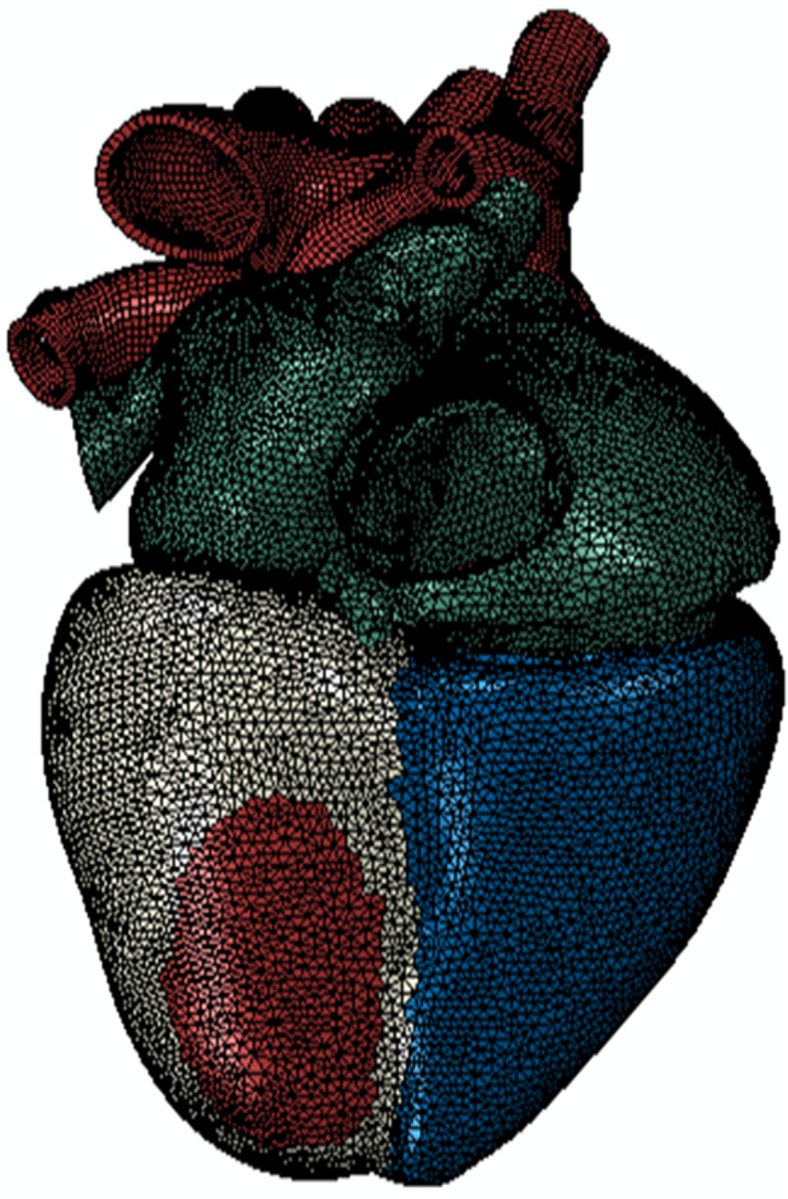
Patient-specific FE model for investigation of treatment of mitral valve regurgitation. Reproduced with permission from Baillargeon et al. [115]

Surgical ventricular restoration (SVR) has been the focus of several computational studies [73–77]. The most recent SVR studies [75, 76] provide examples for how FE models with increased complexity can contribute to elucidating physiological processes and guiding treatment development. For the treatment of MI, Guccione et al. [78] investigated a “myosplint” device aimed at restraining the epicardium to prevent remodelling. Wenk et al. [79] explored the Acorn CorCap cardiac support device as treatment for dilated cardiomyopathy. Another ventricular restoration approach relies on the Parachute© device [80] which was subject of a patient-specific FE study indicating that the reduction in end-diastolic wall stress underlies the therapeutic benefit [43]. Mitral valve regurgitation, a secondary adverse condition resulting from ventricular remodelling, often following MI, has received recent attention with increasingly sophisticated FE models (Fig. 2) [81–84].

## Modelling of material injection therapies for MI

There has been significant recent interest in intramyocardial biomaterial injections as therapy for MI, whereby predominantly the infarcted region of the heart is injected with a biomaterial which aims to inhibit the adverse remodelling that leads to HF. Injectable biomaterials are showing promise in preclinical studies [85–92], resulting in a range of improvements to cardiac repair, with respect to wall thickness, ejection fraction and ventricular volume. Further research on the specific mechanisms by which these biomaterials improve cardiac function is needed to aid the development of more effective treatment.

Research into cardiac injection therapy has become diverse due to the choice of injectable material and the delivery method from a range of viable options [93, 94]. Due to this, the representation of intramyocardial biomaterial injectates in computational models relies on either homogenisation approaches, whereby the injected material is averaged in the description of the myocardium wall, or through geometric approaches, whereby the injections are considered separate inclusions embedded within the wall.

**Fig. 3:**
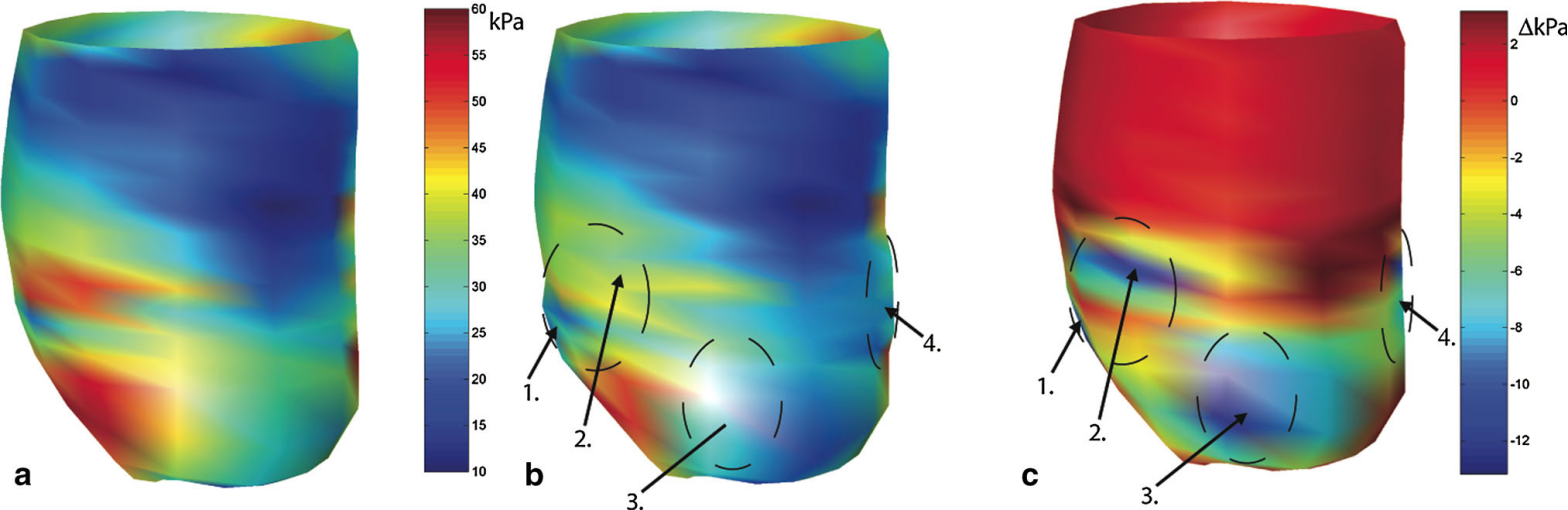
FE prediction of midwall fibre stress in an ovine left ventricle with anteroapical infarct without treatment (**a**) and with simulated intramyocardial delivery 4.4 mL of biomaterial in four infarct border zone locations indicated by arrows (**b**). Difference of midwall fibre stress between the untreated infarct and treated infarct that demonstrates the location of stress reduction in relation to the injection sites (*arrows*) (**c**). Adapted with permission from Wall et al. [95]

**Fig. 4:**
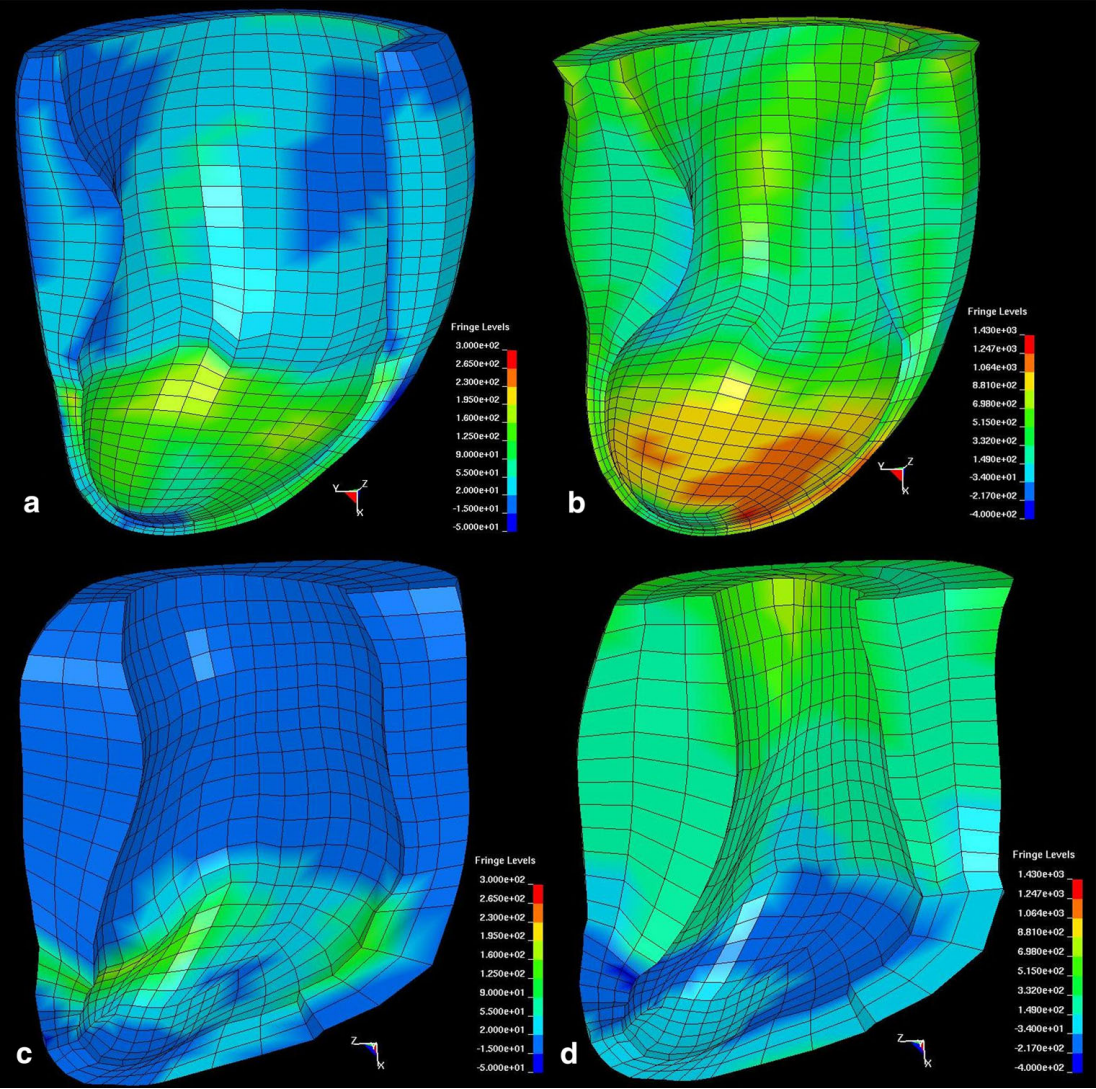
Contour plots of fibre stress in the lateral wall of an ovine left ventricle with untreated infarct at end diastole (**a**) and end systole (**b**), and after treatment by delivery of 2.6 mL of a calcium hydroxyapatite-based tissue filler distributed over 20 evenly spaced injections at end diastole (**c**) and end systole (**d**). (*Colour* scales of the end diastole panels are the same, and colour scales of the end systole panels are the same). Adapted with permission from Wenk et al. [67]

**Fig. 5:**
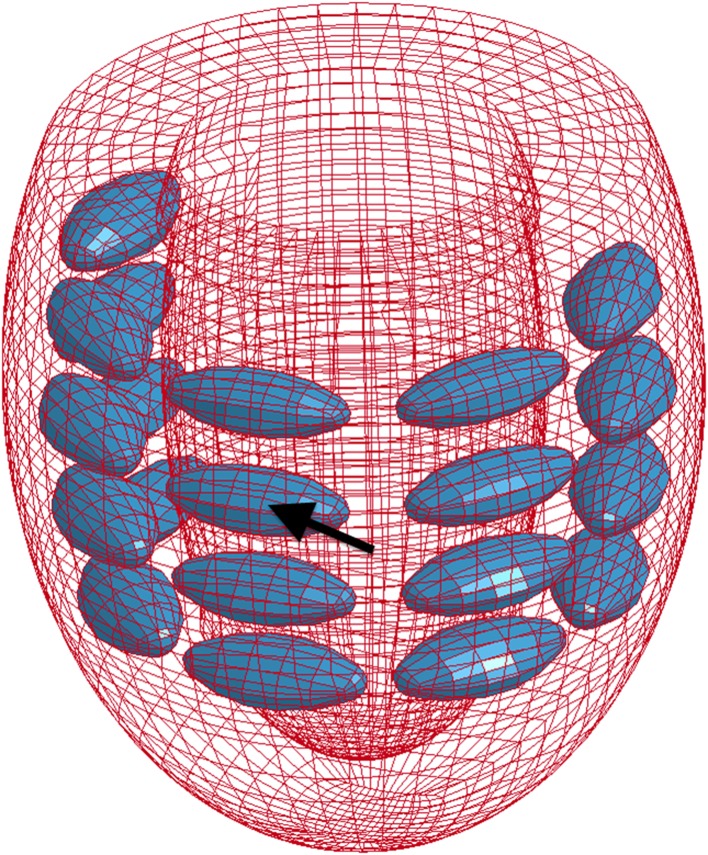
Ellipsoidal LV FE model with 20 intramyocardial hydrogel injectates. Adapted with permission from Kichula et al. [98]

Homogenisation techniques have shown consistently that bulking the myocardium with non-contractile material was sufficient to offset post-MI geometric changes and, consequently decrease stress in the myocardial wall (Fig. 3) [95]. Material injections that result in increased stiffness to the infarct region have also been shown to lower stresses in the infarcted and healthy regions of the heart in subject-specific ovine LV FE models [67] (Fig. 4) and for idealised ellipsoid LV models [96]. Improvements to cardiac function seen in the subject-specific ovine FE model such as wall thickening and increased ejection fraction [67] are consistent with in vitro and in vivo experiments [97]. In a combined experimental and computational study, Kichula et al. [98] used an ellipsoidal LV FE model (Fig. 5) to quantify the anisotropic increase in stiffness due to hydrogel injection and the reduction in local and global wall stresses. Dorsey et al. [92] developed subject-specific porcine LV FE models from cardiac MRI data to estimate the in vivo diastolic material properties of infarcted tissue with therapeutic hyaluronic acid-based hydrogel injections.

**Fig. 6:**
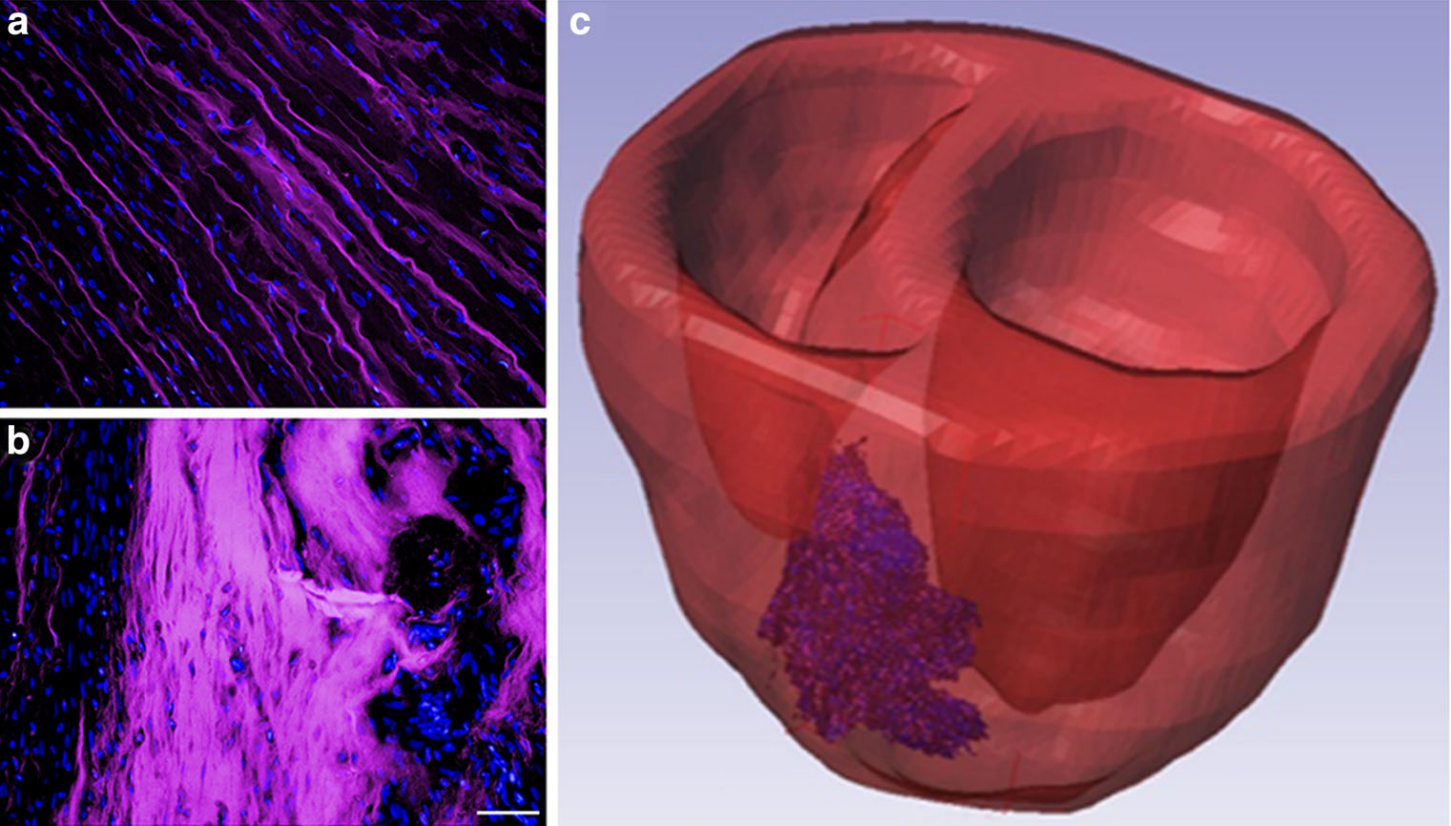
Histological micrographs demonstrating the distribution of a polyethylene glycol hydrogel (appearing in pink) delivered immediately (**a**) and seven days (**b**) after infarct induction in rat hearts (nuclei appear *blue*, *bar* represents 50 μm). Reproduced with permission from Kadner et al. [89]. Reconstructed 3D geometry of a polyethylene glycol hydrogel injectate with microstructural details reconstructed from histological sections in a biventricular rat heart geometry (**c**) (injectate shown in *pink*)

For more viscous injectable materials or when the mechanical effects of the injectate at a microstructural level are being investigated [99–102], modelling the injected material as a discrete inclusion more meaningfully represents the mechanical considerations. Computational studies have consistently shown the beneficial impact to cardiac function from material injections of this nature. Wenk et al. [99] studied in an ellipsoidal LV FE model the optimal distribution of multiple spherical injectates. Kortsmit et al. [101] and Miller et al. [100] modelled the striated and bulk injectate distribution observed preclinically [89, 90, 97], (Fig. 6a, b), as discrete sheet-like structures embedded within the myocardium in a canine biventricular model and a human LV model, respectively. These sheet-like hydrogel inclusions were shown to better improve cardiac performance in the ischaemic infarct stage, but bulk-like injectates were shown to be better at improving LV function at the remodelling stage, complementing an experimental study in rats which investigated the effects of delayed gel-injection therapy [89]. Sirry et al. [103] presented a more realistic microstructurally detailed geometry of a striated polyethylene glycol hydrogel injectate in an infarcted rat heart, similar to Fig. 6c.

**Fig. 7:**
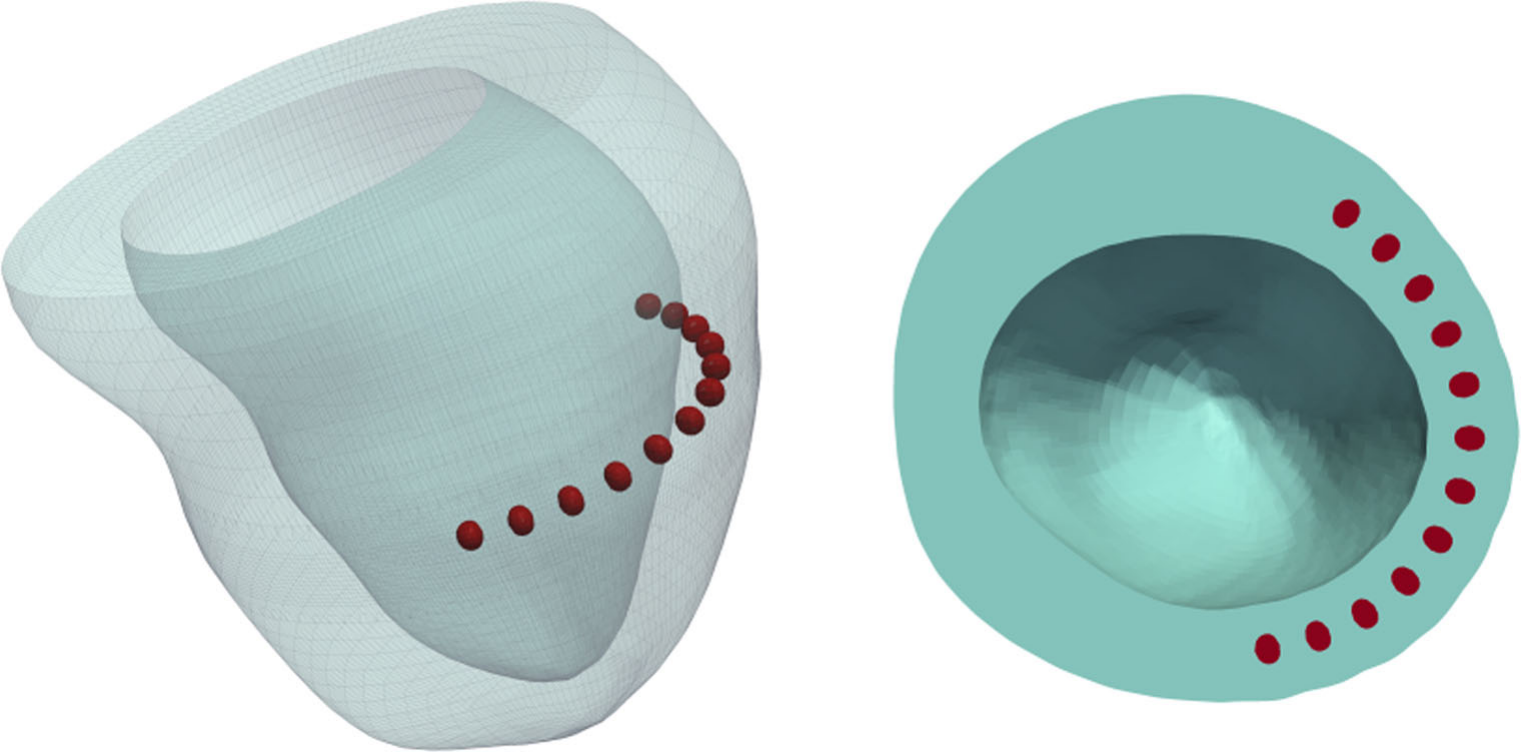
Patient-specific LV FE model with 12 ellipsoidal hydrogel injectates located equidistant between the base and the apex. Adapted with permission from Lee et al. [104]

Residual stress in the cardiac wall due to material injections has only recently been considered. Using a patient-specific LV FE model based on MRI data of a patient with HF, ischaemic cardiomyopathy and hypertension, Lee et al. [104] revealed a complex regional stress field in vicinity of the a set of spherical hydrogel injectates located equidistant between the base and the apex of the LV (Fig. 7). These first results warrant further investigation into the local changes the injections cause to tissue and fibre structure, as well as the mechanisms responsible for the clinically observed reduction in global stress [105].

Lee et al. [106] investigated a combination treatment of biomaterial injections and coronary artery bypass grafting with patient-specific models developed from MRI data of three patients suffering with HF. Simulating a longitudinal study with pre-treatment and three- and six-months post-treatment time points revealed a more uniform distribution and 35 % reduction in myofibre stress throughout the LV.

## Discussion

Advances in patient-specific computational cardiac mechanics over the last decade have been significant in almost every aspect. The quality of imaging and segmentation techniques coupled with increasing computational resources have allowed for unprecedented growth. Geometrically realistic multi-physics models are becoming the new standard of computational cardiology. As structural and functional data of the heart become more easily available, the calibration and validation of these models becomes more reliable.

Patient-specific models hold promise for personalising diagnosis, treatment planning and therapy design. The example of SVR emphasises that interventions based on accurate patient-specific information have clear advantages over treatments that are not personalised. Large-cohort patient-specific computational studies, simulating treatments in silico, will also be able to unlock novel and statistically meaningful findings for entire patient populations—something that cannot be achieved with a small number of computational models. The ability to re-use computational models, perturb parameters and perform sensitivity studies will not only provide an unprecedented wealth of information in the aid of therapy design but can also accelerate the translation of therapy approaches into the clinical setting.

Despite these advantages, there has yet to be a single high-resolution patient-specific computational cardiac model, constructed and calibrated using data from a single patient. Accounting for patient-specific myofibre structure and calibrating material laws using comprehensive in vivo data are still largely lacking in computational models investigating cardiac mechanics.

While recent computational research on biomaterial injection MI therapies has made substantial progress, more work is needed to further elucidate the mechanisms underlying the benefits observed. The local and global changes in myocardial tissue structure after MI, including necrosis, fibrosis and scar formation, and the representation in computational models are one area that need to receive increased attention. Pending availability of experimental data, advanced numerical methods to model growth [107–109] and tissue healing [110] may be adaptable to computationally describe MI-related “reverse” growth and remodelling. This will allow to study in more detail the effects of biomaterial injectates on tissue changes which may provide additional therapeutic cues. A related challenge is the realistic representation of in situ injectate geometries, in particular when biomaterial infiltrates the myocardium at microscopic level [89, 90, 97]. Also yet unconsidered in computational models are injection therapies with mechanobiological targets such as fibroblast reprogramming [111, 112] and stem cell therapies [113, 114].

## Conclusions

Realistic predictive patient-specific computational models require comprehensive in vivo data for calibration and validation. In the context of cardiac diseases and therapies, current in vivo imaging technologies are not yet advanced enough to provide such patient-specific data as part of the clinical diagnostic modalities. Until cutting-edge modalities such as in vivo cardiac DTMRI become more available in the clinic routine, the pursuit of fully subject-specific computational modelling remains limited to preclinical research, where a richer resource of in vivo and ex vivo data can be utilised. Subject-specific computational modelling can, however, offer great potential to complement experimental research and can play a crucial role in advancing biomaterial injection therapies for MI.
